# Beyond diameter: redefining echocardiography criteria in trials of early PDA therapy

**DOI:** 10.1038/s41372-025-02523-7

**Published:** 2025-12-08

**Authors:** Adrianne R. Bischoff, Paula Dias Maia, Patrick J. McNamara

**Affiliations:** 1 Department of Pediatrics, Division of Neonatology, University of Iowa, Iowa City, IA USA; 2 Department of Internal Medicine, University of Iowa, Iowa City, IA USA

**Keywords:** Health care, Cardiovascular diseases

## Introduction

Despite decades of extensive investigation, the assessment and management of the patent ductus arteriosus (PDA) in preterm infants remain uncertain and controversial. To date, there is no universally accepted definition of a “hemodynamically significant PDA”, with existing studies varying in their reliance on clinical criteria, echocardiography parameters, or combined approaches - each with inherent limitations [1–4]. This heterogeneity extends to PDA screening, appraisal of shunt volume, management, and therapeutics, resulting in variable practices worldwide. Moreover, the relationship between the PDA and neonatal respiratory outcomes such as bronchopulmonary dysplasia, pulmonary vascular disease, pulmonary hypertension (PH), pulmonary hemorrhage, and overall mortality from respiratory failure remains unclear. Contemporary statements from leading pediatric societies and institutional clinical practice guidelines favor non-interventional or conservative approaches to PDA care [5]. These are driven by the recent results of randomized clinical trials (RCTs) and meta-analyses, which demonstrate a lack of clear benefit from available pharmacological treatments to close the PDA within the first 72 h in preterm neonates, along with concerns about potential harm [6–9]. The goal of this commentary is not to undermine the recently published RCTs of early ibuprofen, but to reframe their clinical relevance. While one may conclude that early ibuprofen is both ineffective and harmful, an alternative explanation is that the approach to treatment is based on an imprecise definition of PDA significance. It is the opinion of this group that what should be abandoned is the decision to treat the PDA based on an arbitrary threshold of PDA diameter >1.5 mm; instead, clinicians need to recognize that the most recent RCT data are not applicable to the practice of early intervention based on comprehensive characterization of shunt volume.

## Limitations of current early PDA trials

While RCTs provide the strongest available evidence, interpretation and clinical relevance should be limited to the enrolled population. Nearly two decades ago, our group highlighted the need for comprehensive adjudication of hemodynamic significance to ensure only patients with moderate-high volume shunts, the population at greatest risk of PDA attributable morbidity, are enrolled [10]. Yet, most contemporary RCTs employ a pragmatic design; specifically, patients are enrolled based on a transductal diameter threshold of 1.5 mm without other echocardiography markers of shunt magnitude. Indeed, among 1374 patients enrolled across recent RCTs of early PDA treatment, 1214 (88%) met inclusion criteria solely based on transductal size (Table 1).

**Table 1:** Summary of echo criteria across recent randomized controlled trials on early PDA treatment.

Trial	Population	*N*	Echo criteria
Baby-OSCAR (UK, 2024)	Neonates born 23 + 0 to 28 + 6 weeks GA	653	Large PDA (>1.5 mm and unrestricted left-to-right transductal shunting) within 72 h
BeNeDuctus (NL, 2023)	Neonates born <28 weeks GA	273	PDA with diameter >1.5 mm and transductal left-to-right shunt between 24 and 72 h after birth
The PDA RCT (IE, 2021)	Neonates born <29 weeks GA with	60	PDA severity score ≥ 5.0 (with markers of pulmonary overcirculation and LV diastolic dysfunction)
TRIOCAPI (FR, 2021)	Neonates born 24 + 0 to 27 + 6 weeks GA	288	PDA (diameter > 2.26 – (0.078 × postnatal age in hours)) at 6–12 h of life
SMART PDA Trial Protocol (CN, 2024) [1]	Neonates born <26 weeks GA	Target *n* ≈ 100	Combined clinical + echocardiographic criteria (PDA diameter and physiologic markers including LA:Ao ratio, transductal peak systolic velocity, LV output, and diastolic flow pattern in the descending aorta)

Reliance on ductal diameter alone as a marker of hemodynamic significance is problematic for several physiologic and technical reasons. *First*, PDA diameter measurement is inherently operator-dependent, with considerable inter-observer variability, particularly in centers without Targeted Neonatal Echocardiography (TNE) expertise [11]. Encouragingly, data from the Canadian TNE Consortium have demonstrated that this limitation can be overcome [12]; specifically, Weisz et al. demonstrated excellent reliability for echocardiography indices of PDA size, shunt volume, left heart dimensions and output, and diastolic flow abnormalities in systemic arteries, when performed by trained hemodynamic specialists. These data confirm that multiparametric echocardiography indices are reliably reproducible across centers, addressing a key barrier to their use in multicenter clinical trials. For instance, the ongoing PIVOTAL (Percutaneous Intervention Versus Observational Trial of Arterial Ductus in Low Weight Infants) trial is actively enrolling patients using a standardized and comprehensive multiparametric echocardiography protocol where hemodynamic significance is confirmed by an ECHO core laboratory prior to randomization (ClinicalTrials.gov ID: NCT05547165).

*Second*, according to Poiseuille’s Law *(*Q = ΔP·r⁴/8·η·L*)*, transductal flow is determined not only by the radius but also by the pressure gradient between the aorta and the pulmonary artery, ductal length, and blood viscosity. Hence, diameter alone represents only one of several determinants of shunt volume [13]. *Third*, variability in PDA morphology further influences hemodynamic impact. Classic angiographic descriptions (Krichenko A–E and the preterm ‘Type F’ pattern) illustrate how ductal shape, taper, and length can modify resistance and shunt pattern, even for similar diameters [14, 15]. *Fourth*, multiple echocardiography studies have shown that PDA diameter correlates poorly with physiologic indices of shunt magnitude and systemic hypoperfusion, even when indexed to weight or left pulmonary artery diameter [11, 16, 17]. *Finally*, phase-contrast MRI data by Broadhouse et al. [18] demonstrated that PDA diameter correlates weakly with true shunt volume, with left-to-right shunt fractions ranging from <10% to >70% of left ventricular output among infants with comparable ductal diameters. This underscores the principle that anatomic size does not equate to either physiologic burden or clinical impact.

Collectively, these physiologic and technical limitations highlight that using ductal diameter alone to define “hemodynamic significance” may introduce significant heterogeneity in enrolled patient populations and misleading conclusions. Further, the impact of relying solely on diameter may include obscuring potential treatment benefits in high-risk infants, exposing low-risk infants to the side effects of unnecessary pharmacotherapy, and even leading to incorrect treatment in patients with PDA-dependent systemic or pulmonary circulations (i.e., heart dysfunction or PH) [19].

## Hemodynamic adjudication of the early ductus

The field of Neonatal Hemodynamics has evolved over the past two decades to provide longitudinal physiology-based and disease-targeted care to the sickest and most premature neonates [20]. The implementation of TNE has enhanced the adjudication of the hemodynamic significance of the PDA and lowered the need for surgical ligation without increasing adverse outcomes [21]. An early, comprehensive, and multiparametric echocardiographic evaluation using PDA severity scores provides an alternative method for adjudicating hemodynamic significance and identifying true disease states. This approach has been associated with earlier recognition of moderate to high volume shunts, improved PDA management efficiency, and improved outcomes in single-center experiences [22–24], underscoring the importance of evaluating the ductus comprehensively and longitudinally; however, this approach has not yet been subjected to RCT testing.

Another important aspect is that the PDA trajectory matters more than a single time-point assessment. It is plausible that some of the patients enrolled in prior RCTs likely had low-volume shunts or PDAs destined for spontaneous closure. In both scenarios, ductal patency has a short duration and results in little to no clinical relevance. Therefore, in such cases, unnecessary exposure to PDA-directed therapies -whether pharmacological or procedural – may increase the likelihood of treatment-related harm. Importantly, the only currently available natural history data of early PDA physiology assessed by serial echocardiography (El-Khuffash et al. [24]) shows that a transductal diameter threshold of 1.5 mm does not reliably distinguish between shunts destined for spontaneous closure and those associated with significant derangements in other echocardiographic markers. This reinforces the need to incorporate serial, trajectory-based echocardiography adjudication into trial design to enhance diagnostic precision, reduce misclassification, and more accurately identify infants most likely to benefit from intervention.

## Illustrative data supporting comprehensive and longitudinal PDA assessments

**Fig. 1:**
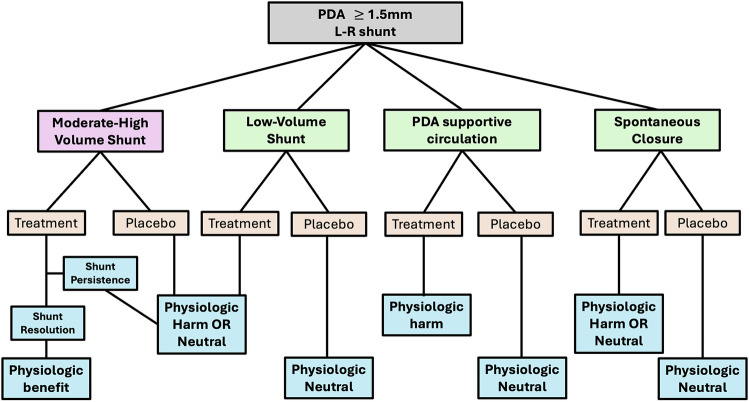
Conceptual framework of pathophysiologic states which may exist in patients with a PDA diameter threshold of ≥1.5 mm. Accurate characterization of the PDA is essential to distinguish scenarios in which closure may confer benefit from those in which it may be neutral or harmful.

The postnatal transition represents a time of major changes in cardiac loading conditions such that the immature myocardium is at increased risk of heart dysfunction and disrupted transition can also cause PH [25]. Therefore, in these cases, ongoing ductal patency may occur and medical therapy aimed at PDA closure would be contraindicated. In addition, ongoing patency may occur in the setting of a high-volume shunt, low-volume shunt or a PDA with high likelihood of spontaneous closure. Figure 1 represents the variable pathophysiologic states that may be associated with ongoing ductal patency. Characterization of the role of the PDA is imperative to distinguish disease states, in which treatment may be beneficial, from scenarios in which PDA closure may be harmful or not necessary. Most RCTs have enrolled babies based on a threshold diameter of 1.5 mm and shunt directionality but there was limited characterization of shunt volume or exclusion of scenarios where PDA closure may be harmful. This reflects a broader gap in the literature, as there are limited data to characterize the biological role of the PDA in the early transitional period.

**Fig. 2:**
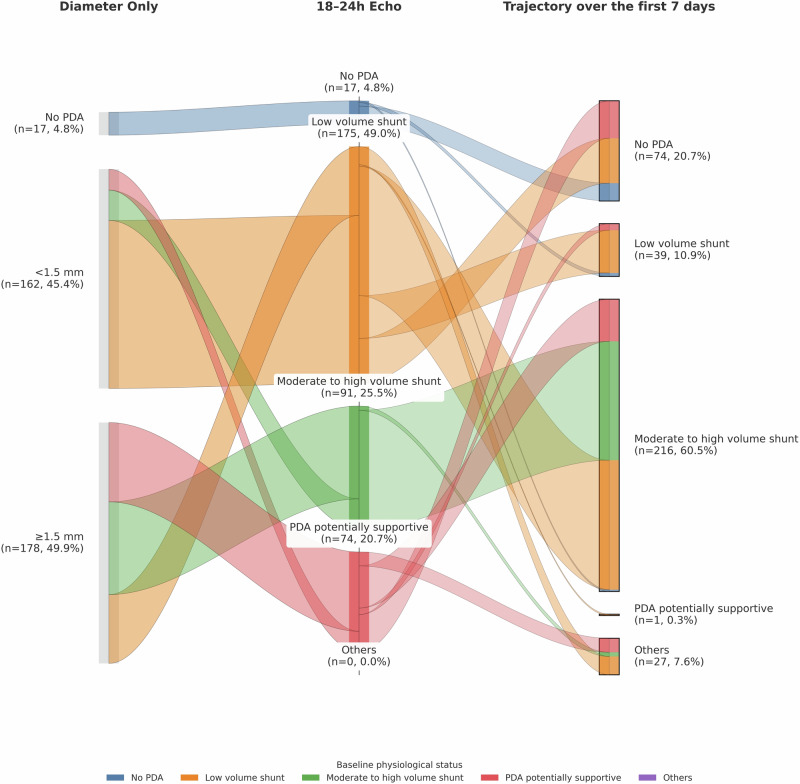
Subgroup analysis of hemodynamic screening target neonatal echocardiograms (TNE) performed in infants born at ≤26 6/7 weeks’ gestation without congenital heart disease at the University of Iowa NICU (January 2019–June 2025). The first column shows classification based on ductal diameter alone (<1.5 mm vs ≥1.5 mm vs no PDA). The second column reflects categorization using the Iowa PDA severity score at 18–24 postnatal hours, and the third column depicts reclassification over the first 7 postnatal days. Flow widths represent the number of infants transitioning between categories. Colors correspond to baseline shunt volume severity (blue: no PDA; orange: low volume shunt; green: moderate–high volume shunt for which medical therapy is recommended; red: supportive physiology such as pulmonary hypertension or heart dysfunction). “Others” represents infants who could not be assigned to a single category by day 7 because of premature death, absence of a repeat TNE assessment, or multiple varying physiologies across the first week. This analysis highlights the heterogeneity of early PDA physiology and the dynamic evolution of ductal significance in the first postnatal week.

As proof of concept, we performed a retrospective analysis of all comprehensive TNE evaluations conducted between January 2019 and June 2025 at the University of Iowa Neonatal ICU among infants born at ≤26 6/7 weeks’ gestation without congenital heart disease, assessed both at 18–24 h and 7 ± 2 days postnatal age (Fig. 2). This analysis illustrates the application of a comprehensive, physiology-based, longitudinal framework for PDA assessment using preliminary unpublished data from our center. A total of 357 infants fulfilled inclusion criteria and 178 had a PDA ≥1.5 mm on the first TNE. Therefore, if ductal size alone were used as trial enrollment criterion, nearly half of these infants would have qualified for randomization in most contemporary early PDA trials [8, 9], irrespective of their PDA physiology. However, when assessed using the Iowa PDA severity score [22], only 70 (39.3%) patients had a moderate-to-high volume shunt for which treatment would be appropriate. In addition, 54 (30.3%) patients had a low-volume shunt, and 54 (30.3%) had PDA supportive physiology, in which treatment would be potentially harmful and therefore contraindicated.

By the end of the first week, irrespective of PDA size or initial physiology, 20.7% of the PDAs (*n* = 74) had closed spontaneously and 10.9% (*n* = 39) remained low-volume shunts, so treatment was not indicated. In contrast, 216 infants (60.5%), developed moderate-to-high volume within the first 7 days, for which treatment was indicated. The remaining 28 infants (7.9%) were categorized as ‘Others,’ representing cases that could not be assigned to a single category by day 7 because of premature death, absence of a repeat TNE assessment, or multiple varying physiologies across the first week. Thus, a ≥1.5 mm size threshold alone may be imprecise and not absolutely representative of the population of interest; specifically, this threshold may include some infants with truly pathologic shunts, but also others in whom closure is either physiologically unimportant or potentially harmful.

## Conclusions and future directions

In summary, clinical trials of early medical treatment of the PDA to date have shown no difference in outcomes and have raised concerns about potential harm. This has led to a contemporary decrease in PDA medical treatment and procedural closure in preterm infants, as reported by the Neonatal Research Network [26] and other cohorts. However, the prioritization of pragmatic design over diagnostic precision remains one of the most significant limitations in current early PDA trials. Echocardiography hemodynamic adjudication is a prerequisite for selecting infants with high-risk PDA phenotypes and generating meaningful and reproducible evidence in PDA science.

In a sub-analysis of our center’s cohort, we observed marked heterogeneity in PDA phenotypes in the first postnatal week in extremely premature patients. Within this population, spontaneous PDA closure was frequently observed, consistent with prior natural history studies [27]. Many infants had PDA-dependent physiology (e.g. PH, heart dysfunction), underscoring the importance of multiparametric and nuanced interpretation. Moreover, a large proportion of infants who did not initially require treatment showed different PDA physiology by day 7 on follow-up assessments, highlighting the importance of serial rather than single-point evaluation.

Therefore, we propose that future early PDA treatment RCTs must adopt: (1) structured, physiology-based multivariable echocardiography assessments of ductal significance beyond ductal diameter - as exemplified by the ongoing SMART-PDA Trial protocol [1], which integrates both clinical and multiparametric echocardiography markers to define ductal significance; (2) trajectory-based evaluation with serial standardized echocardiograms; and (3) collaboration with centers that have neonatal hemodynamics expertise and consideration of expert hemodynamic oversight with centralized ECHO core review - such as that implemented in the PIVOTAL trial (ClinicalTrials.gov ID: NCT05547165) - to improve diagnostic precision and population selection.

Importantly, while Neonatal Hemodynamics expertise is not available in most NICUs, feasibility can be enhanced through multicenter collaboration and centralized core-lab adjudication. These strategies ensure that physiology-based trial design is not limited to specialized centers but can be disseminated broadly across diverse practice environments. Until trials embrace physiologic adjudication, the field risks drawing less reliable conclusions that shape practice but may not optimally serve our most vulnerable patients.
